# Polyclonal Hypergammaglobulinemia Associated With Multimorbidity and Malnutrition in an Elderly Man: A Comprehensive Diagnostic Approach

**DOI:** 10.7759/cureus.93676

**Published:** 2025-10-01

**Authors:** Keigo Hirasa, Junya Ohara, Ryuichi Ohta

**Affiliations:** 1 Community Care, Unnan City Hospital, Unnan, JPN; 2 Family Medicine, Matsue Seikyo Hospital, Matsue, JPN

**Keywords:** adrenal insufficiency, chronic obstructive, family medicine, frailty, general medicine, hypergammaglobulinemia, liver cirrhosis, polyclonal gammopathy, pulmonary disease, rural

## Abstract

A 76-year-old man with a history of liver cirrhosis, chronic obstructive pulmonary disease (COPD), secondary adrenal insufficiency, and psychiatric comorbidities presented with severe weight loss and laboratory findings of pancytopenia, hypoalbuminemia, and IgG-predominant hypergammaglobulinemia, raising suspicion for multiple myeloma. However, serum protein electrophoresis, immunofixation, and urinary Bence Jones testing showed no monoclonal protein, and the kappa/lambda (κ/λ) ratio remained normal, indicating polyclonal hypergammaglobulinemia. Further evaluation revealed decompensated liver cirrhosis with hypersplenism, secondary adrenal insufficiency, severe obstructive ventilatory defect from COPD, and nutritional deficiencies. Management included nutritional supplementation, infection control, diuretics, hydrocortisone replacement, and branched-chain amino acids, leading to gradual improvement and discharge with preserved daily function. This case illustrates how multimorbidity, malnutrition, and disuse can mimic malignant conditions, producing hypergammaglobulinemia and weight loss through chronic systemic inflammation. It emphasizes the importance of considering non-malignant causes, especially in frail older patients, and highlights the need for a comprehensive diagnostic and management approach that integrates chronic disease care, nutritional support, and community-based interprofessional collaboration.

## Introduction

Hypergammaglobulinemia is characterized by elevated levels of serum immunoglobulins. When monoclonal, it raises suspicion for conditions such as multiple myeloma (MM), Waldenström’s macroglobulinemia, or monoclonal gammopathy of undetermined significance (MGUS) [1]. Importantly, hypergammaglobulinemia is also frequently encountered in non-neoplastic settings. Polyclonal hypergammaglobulinemia may result from chronic infections, autoimmune diseases, or chronic liver disorders, and it can also be influenced by aging, malnutrition, and prolonged disuse caused by admission and without rehabilitation in medical institutions or communities [1]. These conditions are common in elderly patients and may complicate the clinical picture by mimicking features of plasma cell dyscrasias [2]. Therefore, a systematic approach to evaluating hypergammaglobulinemia is essential not only for ruling out malignancy but also for identifying treatable non-malignant causes.

In MM, hypercalcemia, renal dysfunction, anemia, and bone lesions, the so-called “CRAB” features, are key diagnostic indicators. However, such abnormalities may also be observed in non-malignant conditions associated with polyclonal hypergammaglobulinemia. For example, anemia may be seen in chronic liver disease, renal dysfunction in autoimmune nephritis, and bone loss in long-standing malnutrition, making careful differentiation essential and demanding systemic investigation, such as upper and lower endoscopies and systemic CT [3-5].

We herein report a case of marked hypergammaglobulinemia in an elderly male, which exemplifies the diagnostic challenge of distinguishing polyclonal hypergammaglobulinemia associated with chronic inflammation from plasma cell dyscrasias. The patient had comorbidities including liver cirrhosis, chronic obstructive pulmonary disease (COPD), and adrenal insufficiency, as well as malnutrition and progressive disuse due to lapses in medical follow-up. Although serum protein electrophoresis did not reveal a clear M-protein, chronic inflammation from multiple underlying diseases and associated nutritional decline were considered contributing factors. This case highlights the importance of recognizing multimorbidity and chronic inflammation as potential causes of hypergammaglobulinemia in elderly patients.

## Case presentation

A 76-year-old man presented with marked weight loss, having lost approximately 5 kg over the preceding six months, at the department of general medicine in a rural hospital. He had been attending regular follow-up visits every two months at our departments of gastroenterology and psychiatry, but had discontinued them on his own approximately six months earlier. On the day of admission, he went out for a dental appointment because of longstanding discomfort with an ill-fitting denture. After visiting the hospital, he visited his sister’s house, where his family noted severe emaciation and took him to the emergency department of our hospital.

At the time of presentation, he had no subjective symptoms, but laboratory tests revealed pancytopenia, hypoalbuminemia, and elevated serum IgG levels, prompting hospitalization for further evaluation of hypergammaglobulinemia. His past medical history included hepatocellular carcinoma (HCC) post-resection, compensated liver cirrhosis, alcohol dependence, alcohol-related dementia, hepatic encephalopathy, chronic kidney disease (G3bA1), depression, schizophrenia, and gastroesophageal reflux disease.

Regarding his medication history, esomeprazole 10 mg once daily, amlodipine 5 mg once daily, and olmesartan 10 mg once daily had been discontinued by the patient about six months earlier. In contrast, trazodone 100 mg once daily, zopiclone 10 mg once daily, zolpidem 5 mg once daily, and flunitrazepam 2 mg once daily had been continued through proxy visits to the hospital by his sister-in-law. On admission, his vital signs were as follows: blood pressure 82/42 mmHg, heart rate 76 bpm, temperature 37.2°C, respiratory rate 10 breaths/min, and SpO₂ 95% on room air. Physical examination showed marked emaciation and dry skin. Although the onset was unclear, a productive cough with whitish viscous sputum was observed at admission. No abnormal findings were noted in the chest or abdomen. His body weight at admission was 40.9 kg.

Laboratory data revealed findings suggestive of malnutrition and pancytopenia. Immunoglobulin fractionation showed IgG-dominant hypergammaglobulinemia (Table 1).

**Table 1:** Initial laboratory data of the patient. Cosyntropin stimulation test values represent serum cortisol after synthetic ACTH administration (baseline, 30 minutes, and 60 minutes). Na: sodium; K: potassium; Cl: chloride; Ca: calcium; P: phosphate; Mg: magnesium

Parameter	Level	Reference
White blood cells	2.9	3.5-9.1×10^3^/μL
Red blood cells	2.89	4.10-5.30×10^6^/μL
Hemoglobin	9.7	11.3-15.2 g/dL
Hematocrit	27.8	33.4-44.9%
Mean corpuscular volume	96.0	79-100 fL
Mean corpuscular hemoglobin	33.4	27-33 pg
Mean corpuscular hemoglobin concentration	34.8	32-36 g/dL
Platelet count	11.6	15.8-34.8×104/μL
Neutrophils (%)	61.3	44.0-72.0%
Lymphocytes (%)	25.1	18.0-59.0%
Monocytes (%)	11.4	2.7-7.9％
Eosinophils (%)	1.9	0.0-10.0％
Basophils (%)	0.3	0-0.5％
Serum Na	132	135-150 mEq/L
Serum K	3.8	3.5-5.3 mEq/L
Serum Cl	101	98-110 mEq/L
Serum Ca	8.0	8.5-10.2 mEq/L
Serum P	2.1	2.5-4.5 mEq/L
Serum Mg	1.8	1.6-2.4 mEq/L
Glucose	103	73-109 mg/dL
Total protein	7.9	6.5-8.3 g/dL
Albumin	1.9	3.8-5.3 g/dL
Blood urea nitrogen	16.7	8-20 mg/dL
Creatinine	1.02	0.40-1.10 mg/dL
Estimated glomerular filtration rate	54.5	>60 mL/min/1.73 m²
Total bilirubin	0.5	0.2-1.2 mg/dL
Direct bilirubin	0.1	<0.3 mg/dL
Aspartate aminotransferase	26	8-38 IU/L
Alanine aminotransferase	10	4-43 IU/L
Gamma-glutamyl transpeptidase	11	<50 U/L
Alkaline phosphatase	74	100-325 U/L
Lactate dehydrogenase	346	121-245 U/L
Creatine kinase	10	38-174 U/L
C-reactive protein	5.43	<0.3 mg/dL
Immunoglobulin G	3830	870-1700 mg/dL
Immunoglobulin A	430	110-410 mg/dL
Immunoglobulin M	45	35-220 mg/dL
Free Kappa light chain	171	3.3-19.4 mg/L
Free Lambda light chain	285	5.7-26.3 mg/L
Kappa/Lambda ratio	0.6	0.26-1.65
Bence Jones protein	-	-
Baseline cortisol	8.4	4.5-21.1 μg/dL
30-min cortisol (cosyntropin stimulation test)	12.5	4.5-21.1 μg/dL
60-min cortisol (cosyntropin stimulation test)	13.8	4.5-21.1 μg/dL
Plasma adrenocorticotropic hormone	35.5	7.2-63.3 pg/mL

**Figure 1:**
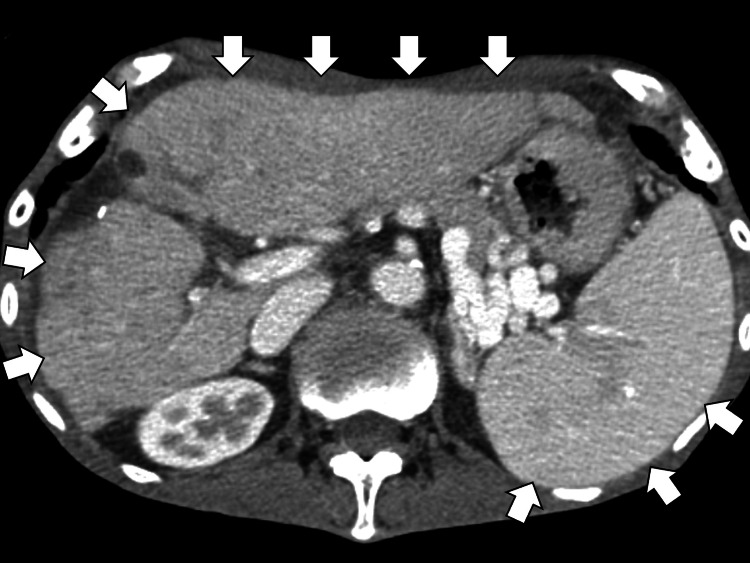
Contrast-enhanced abdominal computed tomography demonstrating splenomegaly in the setting of decompensated liver cirrhosis, suggesting hypersplenism as a potential cause of pancytopenia (white arrows).

On day 5, serum-free κ and λ light chains were assessed, with a κ/λ ratio of 0.6 (reference range: 0.26-1.65). On day 7, urinary Bence Jones protein, hepatitis virus B and C antibodies, and antinuclear antibodies were negative, and immunoelectrophoresis did not reveal a clear M-protein, suggesting a polyclonal immune response. As part of the evaluation for unexplained weight loss, contrast-enhanced CT was performed on day 0 to assess for recurrence of HCC. The scan revealed no evidence of early enhancement or washout suggestive of recurrent HCC. Instead, it demonstrated splenomegaly and irregular liver margins (Figure 1), consistent with decompensated liver cirrhosis and suggesting hypersplenism as a potential cause of pancytopenia.

**Figure 2:**
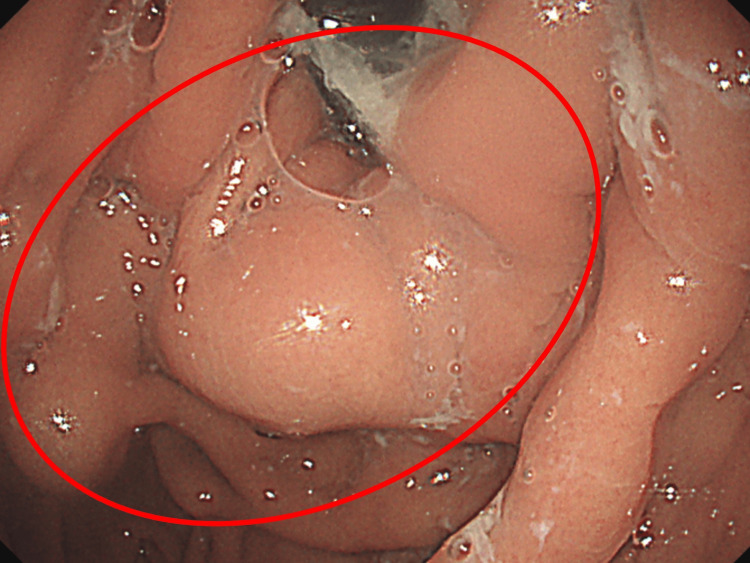
Upper gastrointestinal endoscopy showing gastric varices (a red circle).

Upper gastrointestinal endoscopy on day 6 and colonoscopy on day 8 were performed to rule out malignancy and chronic inflammation. These examinations revealed only gastric varices (Figure 2) and diverticula in the ascending colon, without evidence of malignancy or inflammatory bowel disease.

Because weight loss was accompanied by hypotension (82/42 mmHg at admission), secondary adrenal insufficiency was suspected, and a rapid ACTH stimulation test was performed on day 7. Serum cortisol levels were 8.4 μg/dL at baseline, 12.5 μg/dL at 30 minutes, and 13.8 μg/dL at 60 minutes, while ACTH was within the normal range (35.5 pg/mL) (Table 1). The inadequate peak cortisol response (<18 μg/dL) despite adrenal responsiveness indicated secondary adrenal insufficiency.

**Figure 3:**
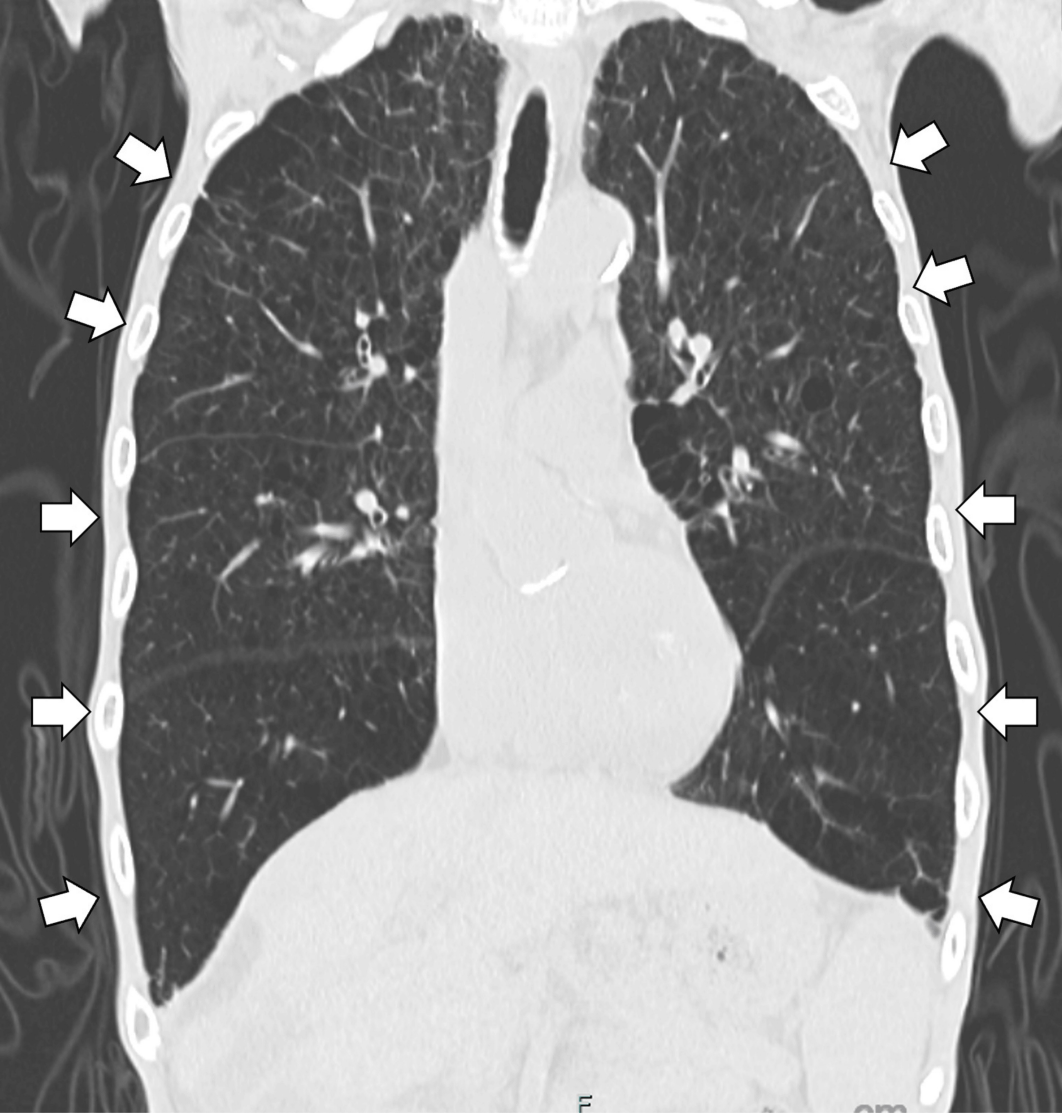
Chest computed tomography revealing emphysematous changes in both lungs, consistent with chronic obstructive pulmonary disease (white arrows).

In addition, since chest CT at admission had demonstrated emphysematous changes, pulmonary function testing was conducted later during hospitalization to assess the contribution of chronic respiratory disease to weight loss and systemic inflammation (Figure 3).

The results showed a severe obstructive ventilatory defect, with FEV₁ 0.99 L (35.4% predicted), FVC 2.22 L (63.2% predicted), and an FEV₁/FVC ratio of 44.6%, leading to a diagnosis of COPD.

Based on these findings, treatment was initiated focusing on nutritional support and management of underlying conditions. From day 0, intravenous acetate Ringer’s solution was administered for volume replacement, and oral supplementation was started for deficiencies identified on admission, including zinc acetate 75 mg/day, folic acid 15 mg/day, and sodium ferrous citrate 50 mg/day. Although hypomagnesemia and hypophosphatemia were also present on admission, supplementation with magnesium oxide 990 mg/day and sodium phosphate 600 mg/day was initiated on day 3.

On day 1, the patient developed hypotension and impaired consciousness, which were attributed to bacterial translocation (BT) in the setting of decompensated cirrhosis and prior HCC. He was treated with cefmetazole 4 g/day until day 5, with no recurrence of symptoms thereafter. On day 6, pitting edema of the dorsum of the feet was observed with dyspnea. The patient was diagnosed with congestive heart failure, and azosemide 30 mg/day was initiated. On the same day, branched-chain amino acids (isoleucine, leucine, and valine) 12.45 g/day were introduced to support protein metabolism in the context of decompensated cirrhosis with hypoalbuminemia. On day 7, secondary adrenal insufficiency was confirmed by rapid ACTH stimulation testing, and oral hydrocortisone 10 mg/day was initiated as replacement therapy. During hospitalization, the patient did not experience recurrent BT. Although oral intake improved to full meals, supplementation of micronutrients remained necessary. Pancytopenia due to liver cirrhosis persisted throughout admission without progression. He was discharged with the previous activities of daily living on day 14 and followed up at an outpatient department in the rural hospital.

## Discussion

This case involved an elderly male with multiple chronic conditions, including liver cirrhosis, COPD, and secondary adrenal insufficiency, who developed malnutrition due to interrupted medical care and progressive disuse and presented with IgG-predominant hypergammaglobulinemia reminiscent of MM. However, immunoelectrophoresis revealed no M-protein, antinuclear antibodies, or hepatitis virus antibodies; the κ/λ ratio was within the normal range, and urinary Bence Jones protein was negative, supporting a diagnosis of polyclonal hypergammaglobulinemia related to chronic inflammation and/or liver disease. Multiple chronic illnesses likely sustained increased immunoglobulin production, while nutritional deficiencies, trace element depletion, and adrenal insufficiency further disrupted immune regulation.

Hypergammaglobulinemia is classified as monoclonal or polyclonal. Monoclonal forms occur in B-cell neoplasms such as MM and MGUS, characterized by M-protein detection [1]. Polyclonal hypergammaglobulinemia arises from persistent antigenic stimulation in chronic infections, autoimmune diseases, and liver disorders, activating multiple B-cell clones to produce immunoglobulins broadly [6]. In chronic inflammation, cytokines such as IL-6 drive B-cell differentiation and plasma cell formation, often causing IgG-predominant increases [7]. In liver cirrhosis, portal hypertension can increase intestinal permeability, promoting BT, which sustains chronic inflammation [8]. Certain commensal Gram-negative bacteria entering systemic circulation via BT can induce IgG production [9], perpetuating elevated inflammatory cytokines and immune cell activation, thereby enhancing polyclonal immunoglobulin production. In COPD, persistent airway inflammation elevates IL-6 and TNF-α, which may also contribute to excessive immunoglobulin production [10]. COPD-related systemic inflammation is increasingly recognized as a driver of comorbidity clusters in elderly patients. Thus, in this case, multiple overlapping chronic diseases likely led to polyclonal hypergammaglobulinemia driven by chronic inflammation.

This patient’s decompensated cirrhosis, COPD, secondary adrenal insufficiency, malnutrition, and disuse acted synergistically to cause physical, metabolic, and immune vulnerability characteristic of frailty. Frailty is not merely a consequence of aging or functional decline; its underlying pathologies promote skeletal muscle atrophy, chronic inflammation, and psychological frailty, which in turn accelerates disuse-related decline, leading to a multi-layered immune dysregulation [11].

In decompensated cirrhosis, portal hypertension increases gut permeability, allowing BT to activate immune cells and inflammatory cytokine production, sustaining systemic inflammation [12].

In COPD, persistent airway inflammation with chronically elevated IL-6 and TNF-α promotes protein degradation via the ubiquitin-proteasome system, muscle fiber type shifts, and mitochondrial dysfunction [13], resulting in sustained systemic inflammation, heightened infection susceptibility, and qualitative changes in immune responses. Secondary adrenal insufficiency impairs cortisol-mediated suppression of inflammatory cytokines and immune regulation, allowing IL-6 and TNF-α to remain elevated, further accelerating skeletal muscle protein breakdown [14].

Malnutrition limits the supply of essential amino acids for muscle synthesis and, together with hypoalbuminemia and trace element deficiencies (zinc and folate), impairs muscle anabolism and immune modulation [15]. This also reduces oxidative stress tolerance and hematopoiesis, weakening immune defenses and making inflammation harder to control. In this case, frailty fostered a vicious cycle of muscle loss, chronic inflammation, and immune dysfunction, contributing to the background of hypergammaglobulinemia and signaling long-term physiological decline. A comprehensive assessment of immune dysfunction in older adults is essential for holistic management [16]. To improve their frailty, general physicians should support older individuals with multimorbidity by involving them in community activities through interprofessional collaboration within medical institutions and the broader community [17-19].

## Conclusions

This case highlights that hypergammaglobulinemia in elderly patients is not always attributable to hematologic malignancy but can also arise from chronic inflammation associated with multimorbidity, liver cirrhosis, COPD, adrenal insufficiency, and malnutrition. The overlapping effects of these conditions contributed to polyclonal immunoglobulin elevation that initially mimicked plasma cell dyscrasia. Careful evaluation with serum protein studies and a holistic assessment of underlying chronic diseases were essential to avoid misdiagnosis. General physicians should consider polyclonal hypergammaglobulinemia as a manifestation of chronic systemic inflammation in frail older patients and address not only disease-specific management but also nutritional support, functional rehabilitation, and interprofessional collaboration to improve long-term outcomes.

## References

[1] Zhao EJ, Cheng CV, Mattman A, Chen LYC (2021). Polyclonal hypergammaglobulinaemia: assessment, clinical interpretation, and management. Lancet Haematol.

[2] Marinkovic A, Zypchen LN, Chan J, Chen LYC, Parkin S (2022). Monoclonal gammopathy of clinical significance: what the rheumatologist needs to know. Lancet Rheumatol.

[3] Manrai M, Dawra S, Kapoor R, Srivastava S, Singh A (2022). Anemia in cirrhosis: an underestimated entity. World J Clin Cases.

[4] Kronbichler A, Mayer G (2013). Renal involvement in autoimmune connective tissue diseases. BMC Med.

[5] Montalcini T, Romeo S, Ferro Y, Migliaccio V, Gazzaruso C, Pujia A (2013). Osteoporosis in chronic inflammatory disease: the role of malnutrition. Endocrine.

[6] Kyle RA, Rajkumar SV (2009). Criteria for diagnosis, staging, risk stratification and response assessment of multiple myeloma. Leukemia.

[7] Grebenciucova E, VanHaerents S (2023). Interleukin 6: at the interface of human health and disease. Front Immunol.

[8] Nishimura N, Kaji K, Kitagawa K (2021). Intestinal permeability is a mechanical rheostat in the pathogenesis of liver cirrhosis. Int J Mol Sci.

[9] Zeng MY, Cisalpino D, Varadarajan S (2016). Gut microbiota-induced immunoglobulin G controls systemic infection by symbiotic bacteria and pathogens. Immunity.

[10] Xu J, Zeng Q, Li S, Su Q, Fan H (2024). Inflammation mechanism and research progress of COPD. Front Immunol.

[11] Evans WJ, Paolisso G, Abbatecola AM, Corsonello A, Bustacchini S, Strollo F, Lattanzio F (2010). Frailty and muscle metabolism dysregulation in the elderly. Biogerontology.

[12] Wiest R, Lawson M, Geuking M (2014). Pathological bacterial translocation in liver cirrhosis. J Hepatol.

[13] Jaitovich A, Barreiro E (2018). Skeletal muscle dysfunction in chronic obstructive pulmonary disease. What we know and can do for our patients. Am J Respir Crit Care Med.

[14] Webster JM, Kempen LJ, Hardy RS, Langen RC (2020). Inflammation and skeletal muscle wasting during cachexia. Front Physiol.

[15] Balamurugan BS, Marimuthu MM, Sundaram VA, Saravanan B, Chandrababu P, Chopra H, Malik T (2024). Micro nutrients as immunomodulators in the ageing population: a focus on inflammation and autoimmunity. Immun Ageing.

[16] Ohta R, Yakabe T, Sano C (2024). Frailty syndrome in rural communities: a narrative review and interviews with rural individuals. Cureus.

[17] Ohta R, Nitta T, Shimizu A, Sano C (2024). Role of family medicine physicians in providing nutrition support to older patients admitted to orthopedics departments: a grounded theory approach. BMC Prim Care.

[18] Ohta R, Yoshioka K, Sano C (2024). Evolution of the roles of family physicians through collaboration with rehabilitation therapists in rural community hospitals: a grounded theory approach. BMC Prim Care.

[19] Ohta R, Sano C (2023). The effectiveness of family medicine-driven interprofessional collaboration on the readmission rate of older patients. Healthcare (Basel).

